# Antidepressants use during pregnancy and child psychomotor, cognitive and language development at 2 years of age—Results from the 3D Cohort Study

**DOI:** 10.3389/fphar.2023.1252251

**Published:** 2023-11-16

**Authors:** Noémie Tanguay, Nadia Abdelouahab, Marie-Noelle Simard, Jean R. Séguin, Isabelle Marc, Catherine M. Herba, Andrea A. N. MacLeod, Yohann Courtemanche, William D. Fraser, Gina Muckle

**Affiliations:** ^1^ École de psychologie, Université Laval, Québec, QC, Canada; ^2^ Centre de Recherche du CHU de Québec-Université Laval, Québec, QC, Canada; ^3^ Centre de Recherche du CHUS de Sherbrooke, Sherbrooke, QC, Canada; ^4^ Centre de Recherche du CHU Sainte-Justine, Montréal, QC, Canada; ^5^ École de réadaptation, Université de Montréal, Montréal, QC, Canada; ^6^ Département de psychiatrie et d’addictologie Université du Québec à Montréal, Montréal, QC, Canada; ^7^ Département de pédiatrie, Université Laval, Québec, QC, Canada; ^8^ Département de psychologie, Université du Québec à Montréal, Montréal, QC, Canada; ^9^ Department of Communication Sciences, University of Alberta, Edmonton, AB, Canada

**Keywords:** antidepressants, pregnancy, child development, cognition, motor skills, language

## Abstract

**Introduction:** Approximately 5.5% of pregnant women take antidepressants. Studies on prenatal exposure to antidepressants reported no association with child cognition, and inconsistent results with motor function and language development. A limitation has been the failure to adjust for prenatal maternal distress.

**Objectives:** Assess the associations between prenatal exposure to antidepressants and child development at age two, while adjusting for maternal depressive symptoms and stress during pregnancy. Explore indirect effects through birth complications and consider sex-specific associations.

**Methods:** This is an ancillary study of the 3D (Design Develop, Discover) Study initiated during pregnancy. Data on antidepressants were collected through medication logs spanning the entire pregnancy. Depressive symptoms and stress were assessed during pregnancy by self-reported questionnaires, motor and cognitive development with the Bayley Scales of Infant and Toddler Development (BSID-III), and language development with the MacArthur Communicative Development Inventories at age 2. Multiple linear regressions were used to assess the associations between exposure and developmental outcomes. Mediation models were used to assess indirect effects. Interaction terms were introduced to assess sex-specific associations.

**Results:** 1,489 mother-child dyads were included, of whom 61 (4.1%) reported prenatal antidepressant use. Prenatal exposure was negatively associated with motor development (*B* = −0.91, 95% CI -1.73, −0.09 for fine motor, *B* = −0.89, 95% CI -1.81, 0.02 for gross motor), but not with cognitive (*B* = −0.53, 95% CI -1.82, 0.72) and language (*B* = 4.13, 95% CI -3.72, 11.89) development. Adjusting for maternal prenatal distress only slightly modified these associations. No indirect effect or differential effect according to child sex were found.

**Conclusion:** This study supports evidence of a negative association between prenatal exposure to antidepressants and motor development at age two, after adjusting for maternal distress, but the effect size remains very small, with about only one BSID-III point lower in average.

## Background

Depression is characterized by depressed mood, loss of interest or pleasure, feelings of worthlessness, and/or weight, sleep, cognitive and/or behavioural changes (American Psychiatric Association, 2015). In pregnant women, a systematic review based on information available in 2019 estimates the worldwide prevalence of depression at 12.6%, as established by a structured clinical interview, but elevated depressive symptoms are reported by 22.5% when based on self-reported questionnaires (Yin et al., 2021).

When treating depression in pregnant women, it is necessary to consider the potential risks to the pregnancy and the foetus. The American Psychiatric Association and the Canadian Network for Mood and Anxiety Treatments clinical guidelines recommend that psychotherapy must be considered as first-line treatment, independently of the depression severity, and pharmacotherapy should be considered a second-line recommendation (American Psychiatric Association, 2010; MacQueen et al., 2016). The prevalence of antidepressant use during pregnancy is estimated at 5.5% in North America (Molenaar et al., 2020). In Quebec, for pregnancies occurring between 1998 and 2002, 3.7% of pregnant women took antidepressants during the first trimester, 1.6% during the second trimester, and 1.1% during the last trimester (Ramos et al., 2007).

Antidepressants traverse the placental barrier and may affect the foetus. SSRIs (selective serotonin reuptake inhibitors), the most commonly prescribed antidepressant, have been associated with increased extracellular serotonin concentrations (Rampono et al., 2009), potentially altering the development of the serotonergic circuitries of the foetus, leading to reduced endogenous production (Oberlander et al., 2009). Serotonin plays a major role in the developing brain and lower concentrations of endogenous serotonin could affect cognition, learning abilities and stress response (Gaspar et al., 2003). The serotonergic system is also involved in muscle tone and motor performances (Galbally et al., 2012).

Studies examining associations between prenatal exposure to antidepressant medications and birth outcomes provide empirical evidence of a greater risk for preterm births (Fitton et al., 2020; Vlenterie et al., 2021), inconsistent results with birthweight (Tak et al., 2017; Fitton et al., 2020), and a possible increased risk of cardiac malformations for paroxetine and fluoxetine (Fitton et al., 2020).

Evidence supporting negative associations with child development before 4 years of age are less abundant. Regarding motor development, two systematic reviews found no evidence of association between prenatal exposure to antidepressants and child development, or inconsistent results since negative, weak positive and absence of associations are all reported (Araujo et al., 2020; Al-Fadel and Alrwisan, 2021). One review however found a significant negative association (Prady et al., 2018). A meta-analysis was conducted and included studies published up to 07/2017 reporting primary data (excluding individual case studies) and including a control group. The results support significant negative associations with an overall occurrence of poorer motor outcomes [effect size (standardized mean difference) = 0.22, 95% CI 0.07, 0.37] (Grove et al., 2018). Regarding cognitive development, all three systematic reviews concluded no evidence of association with prenatal antidepressants (Prady et al., 2018; Araujo et al., 2020; Al-Fadel and Alrwisan, 2021). For language development, only one systematic review included more than one study with a language outcome and found no evidence of an association (Araujo et al., 2020).

However, an important limitation reported by authors is the absence of adjustment for prenatal maternal distress. Maternal depression, anxiety, and stress during pregnancy have all been shown to be negatively associated with offspring cognitive, motor, and language development (Rogers et al., 2020; Martucci et al., 2021) and it is expected that women taking antidepressants suffered from psychological distress. However, only a few studies of prenatal exposure to antidepressants have adjusted for prenatal maternal distress. For motor development, half of these studies reported negative associations [*OR* = 1.42, 95% CI 1.07-1.87 for being in a lower development category on the Age and Stages Questionnaire (Handal et al., 2016), and a mean difference of 1.1–1.2 points on the BSID gross motor scale (van der Veere et al., 2020)], while other studies did not (Nulman et al., 2002; Gustafsson et al., 2018). For cognitive development, most studies reported nonsignificant results (Nulman et al., 2002; Nulman et al., 2012; Johnson et al., 2016; Gustafsson et al., 2018) and only one reported a negative association (van der Veere et al., 2020). For language development, when adjusting for maternal distress, all studies obtained significant, but conflicting results (Nulman et al., 2002; Skurtveit et al., 2014; Johnson et al., 2016).

Our examination of the primary studies included in those systematic reviews and meta-analysis, and of more recent studies (Gustafsson et al., 2018; Galbally et al., 2021), lead to the observations that negative associations with child development outcomes are 1) mostly found in large sample size studies―most studies have limited statistical power to detect small to moderate associations due to small samples―, 2) when outcomes are reported by the mother, and 3) very few studies adjust for maternal distress during pregnancy, which can be associated with both the use of antidepressant and child developmental outcomes. Some of these limitations have already been highlighted (Araujo et al., 2020; Al-Fadel and Alrwisan, 2021).

The objective of this study is to assess the direct associations between prenatal exposure to antidepressants and child motor, cognitive and language development at 2 years of age, while adjusting for maternal distress during pregnancy through self-report of depressive symptoms and perceived stress. Secondary exploratory objectives include the examination of 1) an indirect effect through duration of gestation and birthweight, and 2) if the hypothesized associations differ by child sex since there is accumulating empirical evidence for sex difference vulnerabilities in studies focusing on developmental effects of utero exposure to environmental pollutants (Oulhote et al., 2020) and prenatal maternal distress (Sutherland and Brunwasser, 2018; Kumpulainen et al., 2023; Wu et al., 2023), and strong biological plausibility given the sexually dimorphic nature of prenatal development.

## Methods

### Study design and sample

**FIGURE 1:**
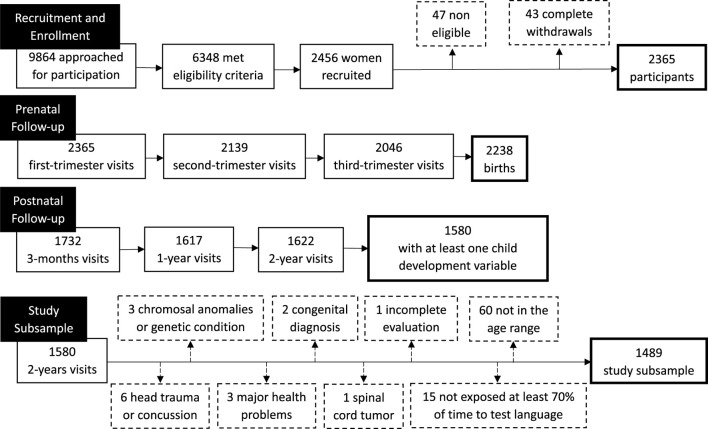
Flowchart for 3D Cohort Study participants included in the present study.

This project is an ancillary study of the 3D Cohort Study, a prospective study of Quebec’s pregnant women recruited during the first trimester (Fraser et al., 2016). It comprises data collected in case report form by research nurses, abstracted from obstetrical and neonatal hospital records, reported by mothers each trimester of pregnancy and postnatally through self-administered questionnaires, and direct child assessments carried out at one of the study centres. Women had to be between 18 and 47 years of age at the time of recruitment, and able to communicate in French or English. Exclusion criteria included current intravenous drug use, severe illness or life-threatening conditions, and multiple gestation pregnancies. Between May 2010 and August 2012, 2,366 women were recruited and followed in one of the nine collaborating hospital (see Fraser et al. (2016) for details). The 2-year follow-up comprises 1,580 families with an in-person child development evaluation (last follow-up March 2015). For our ancillary study, additional inclusion criteria included that the child be tested between 18 and 30 months of age, and a language development score obtained from French, English or Spanish test version from children being exposed to the test language at least 70% of the time. Children with medical conditions known to interfere with child development were excluded from our analyses. Our final sample includes 1,489 mother-child dyads (Figure 1), with a sample size for analyses ranging from 1,055 to 1,456 according to the developmental outcome (Supplementary Figure S1).

### Measures

Use of antidepressants was collected at each trimester of pregnancy through a maternal medication log, and recorded via interviewer-administered questionnaires for the 3 months prior to pregnancy. Exposure was treated as a yes/no use of at least one antidepressant at any moment during pregnancy. Prenatal questionnaire at first trimester provided socio-demographic information including marital status (married or living with someone vs. not), annual family income in Canadian dollars (below 40,000$, 40,000$–79,999$, higher than 80,000$), maternal age (years), education (number of years) and parity before this pregnancy. Cigarette smoking and alcohol consumption (yes/no) were documented at each trimester of pregnancy. Gestation duration (weeks), birthweight (gr.), child sex were collected from hospital medical records. Child age (years) at time of testing was derived from dates of birth and of testing. Maternal distress during pregnancy was documented with two well-validated self-reported questionnaires: 1) the Center for Epidemiologic Studies—Depression Scale (Radloff, 1977) [CES-D - 10 items; Cronbach’s *α* = .89 (Björgvinsson et al., 2013)] completed at the first trimester of pregnancy, 2) the Perceived Stress Scale (PSS-4, Cronbach’s *α* = 0.79 (Karam et al., 2012; Cohen et al., 1983) completed during the second trimester.

Cognitive, fine and gross motor development were assessed at 2-years with the Bayley Scales of Infant and Toddler Development—Third Edition (BSID-III), administered in each research site by research nurses and research assistants dedicated to this project and trained by one licensed psychologist who was also responsible for post-training quality control. BSID-III items are rated 0 (skill not acquired) or 1 (skill acquired), and the subscales comprise 91, 66 and 72 items, respectively (Albers et al., 2006). A short version of the expressive vocabulary scale of the MacArthur Communicative Development Inventories (MCDI) (level II—form A) was handed over to the parent, mostly the mother, who returned the questionnaire by post or Internet; the respondents were asked to indicate the words said by the child on a list of 100 words (Fenson, 1993; Brodeur et al., 2016). The validity of the BSID-III is well established on general populations as the 3D cohort, and correlations of BSID-III scores with socioeconomic indicators, maternal distress, child sex, birthweight, gestation duration and MCDI score presented in Table 2 are providing information on its convergent validity in our sample. Higher BSID-III and MCDI scores reflected a more favourable development.

### Statistical analyses

Comparative analyses (Welch’s *t*-test, chi-Square test of independence and Wilcoxon’s rank sum test) were carried out to compare the 3D participants included and not included in the present study. A DAG (directed acyclic graph) was developed to identify the confounders to be included in multivariate analyses (Shrier and Platt, 2008), from the list of the following potential variables selected for their hypothesized causal links based on previous studies and systematic reviews: child’s sex and age at testing, maternal age and parity at delivery, socioeconomic status (maternal education and annual family income), psychological distress (maternal depression and perceived stress), and substance use. We retained child’s sex and age at testing, maternal age, stress and depressive symptoms as mandatory covariates, as well as maternal education and annual family income as they were outlined by the DAG (see Supplementary Figure S2). Multiple linear regressions were used to assess the associations between prenatal exposure and child development, and four models were tested for each outcome: 1) minimally adjusted (child’s sex and age at testing, maternal age and education, annual family income); 2) adjusting for first trimester maternal depressive symptoms only; 3) adjusting for second trimester maternal stress only; and 4) fully adjusted for both depressive and stress symptoms. Fully adjusted mediation models were used to explore indirect effects explained by duration of gestation and birthweight, and interaction terms were introduced to assess differential effects according to child sex (moderation analysis). The E-value was estimated using VanderWeele and Ding (VanderWeele and Ding, 2017) method to address unmeasured confounding. Analyses were conducted using IBM SPSS Statistics 26 and R 4.1.0 (Rosseel, 2012; Kowarik and Templ, 2016; Team, 2022), and regressions were conducted using full information maximum likelihood estimation and bootstrapping (Lee and Shi, 2021).

### Ethics approval

The 3D Cohort protocol and procedures were approved by the Research Ethics Committees at the coordinating centre (Montreal CHU Sainte-Justine, approbation MP-21-2010-233) and in all study sites.

## Results

Sample characteristics are presented in Table 1. The study sample was largely composed of women in their late twenties/early thirties, living with a partner, highly educated and with a high annual family income. Few participants reported using tobacco during pregnancy, while more than half reported using alcohol. Few children were born preterm (5.4% < 37 weeks of gestation) or were low birthweight (3.2% < 2,500 g), and half were girls. Maternal antidepressant use during pregnancy was reported by 4.1% of the participants who most often used it daily (96.2%), and for an average duration of 6 months. Since less than 1 in 20 women reported antidepressant use, exposure was treated as yes/no use of at least one antidepressant at any moment during pregnancy. Characteristics of the 3D participants not included in the present study are presented in Supplementary Table S1, which showed that our participants had a higher annual income, were more likely to use alcohol and less likely to use tobacco while pregnant. BSID-III and MCDI scores were higher for the excluded participants, but children were also older at testing in this group.

**TABLE 1:** Characteristics of study participants.

Variable	N	Mean ± SD or *n* (%)	Range
Family and maternal characteristics			
Marital status (% married or living with someone)	1,488	1,427 (95.9)	
Annual income (in CDN$)	1,431		
<40,000$		189 (13.2)	
40,000$ - 79,999$		425 (29.7)	
≥80,000$		817 (57.1)	
Mother’s education (years)	1,463	16.85 ± 3.07	6.00–30.00
Parity before childbirth	1,463	0.58 ± 0.81	0.00–10.00
Maternal age at delivery (years)	1,486	32.14 ± 4.42	18.39–47.70
Maternal stress 2nd trim. of pregnancy^a^	1,347	3.44 ± 2.80	0.00–16.00
Maternal depression 1st trim. of pregnancy^b^	1,209	7.16 ± 4.52	0.00–27.00
Maternal tobacco use during pregnancy (% yes)	1,489	145 (9.7)	
Maternal alcohol use during pregnancy (% yes)	1,488	818 (55.0)	
Prenatal exposure to antidepressant			
Maternal use (% yes)	1,489	61 (4.1)	
Daily use during pregnancy (%)	53	51 (96.2)	
Duration of use (in months)	61	5.51 ± 3.20	1.00–9.00
Child characteristics			
Child sex (% girls)	1,489	743 (49.9)	
Gestation duration (weeks)	1,477	38.98 ± 1.60	27.00–42.00
Birthweight (kg)	1,487	3.39 ± 0.51	0.96–5.53
Age at BSID-III assessment (months)	1,460	24.77 ± 1.76	22.00–30.00
Age at MCDI assessment (months)	1,055	24.74 ± 1.76	20.00–30.00
Child outcomes			
Cognition raw score—BSID-III	1,456	63.97 ± 5.40	39.00–79.00
Fine motor skills raw score– BSID-III	1,397	41.22 ± 3.43	29.00–54.00
Gross motor skills raw score– BSID-III	1,397	55.09 ± 3.65	43.00–65.00
Language raw score—MCDI	1,055	55.36 ± 22.99	2.00–100.00

Note. BSID-III, Bayley Scales of Infant and Toddler Development–Third Edition; MCDI, MacArthur-Bates Communicative Development Inventories.

^a^ Perceived Stress Scale—4 items.

^b^ Center for Epidemiologic Studies–Depression Scale—10 items.

Missing data follows a MNAR pattern (missing not at random), and the three multivariate missing data patterns with the highest proportion of missing values are depressive symptoms missing (12.0%), both depressive symptoms and stress missing (5.1%), stress missing (3.9%) (see Supplementary Figure S3).

Correlations between study variables are presented in Table 2. Strongest intercorrelations were found among developmental indicators (*r's*: 0.31–0.58), gestation duration and birthweight (*r* = 0.56), depressive symptoms and perceived stress (*r* = 0.44). Antidepressant exposure was marginally associated with fine motor (*r* = −0.05), gross motor (*r* = −0.04) and cognitive development (*r* = −0.03), but not with language. Antidepressant exposure was related to maternal depressive symptoms (*r* = 0.11) and perceived stress (*r* = 0.16). Maternal perceived stress was related to cognitive (*r* = −0.11), fine motor (*r* = −0.07) and language (*r* = −0.09) development, but not with gross motor, while depressive symptoms were not associated with any of the child outcomes (*r's*: −0.04 to 0.04). Gestational age was associated with cognitive, fine motor and language development (*r's*: 0.06–0.12), but not with gross motor, while birthweight was related to cognition, fine motor and gross motor (*r's*: 0.06–0.08), but not with language. Gestational age and birthweight were not associated with antidepressant use.

**TABLE 2:** Correlations of study variables.

Variable	1	2	3	4	5	6	7	8	9	10	11	12	13	14	15
1. Antidepressant exposure^a^	‒														
2. Cognition—BSID-III^a^	−.03 (−.08, .02)	‒													
3. Fine motor skills—BSID-III^a^	−.05 (−.10, .00)	.58 (.54, .61)	‒												
4. Gross motor skills—BSID-III^a^	−.04 (−.10, .01)	.41 (.36, .45)	.41 (.37, .45)	‒											
5. Language—MCDI^a^	.02 (−.03, .08)	.51 (.47, .55)	.36 (.32, .41)	.31 (.26, .36)	‒										
6. Child sex^a^ ^,^ ^b^	.02 (−.03, .07)	.11 (.06, .16)	.16 (.10, .21)	.05 (.00, .11)	.16 (.10, .21)	‒									
7. Child age at BSID-III testing^a^	.01 (−.05, .06)	.41 (.36, .45)	.43 (.39, .47)	.31 (.26, .36)		−.01 (−.06, .04)	‒								
8. Child age at MCDI assessment^a^	.03 (−.02, .09)				.28 (.23, .33)	.01 (−.05, .06)	.89 (.88, .90)	‒							
9. Perceived stress^a^	.11 (.06, .16)	−.11 (−.16, −.06)	−.07 (−.12, −.01)	−.01 (−.07, .04)	−.09 (−.15, −.04)	−.00 (−.06, .05)	.06 (.00, .11)	.07 (.01, .12)	‒						
10. Depressive symptoms^a^	.11 (.05, .17)	−.02 (−.08, .04)	.04 (−.01, .10)	.04 (−.02, .10)	−.04 (−.10, .02)	.03 (−.02, .09)	.03 (−.03, .09)	.08 (.02, .14)	.44 (.39, .48)	‒					
11. Maternal anxiety^a^	.10 (.04, .16)	−.07 (−.12, −.01)	−.03 (−.08, 04)	−.03 (−.09, .03)	−.03 (−.09, .03)	.01 (−.05, .06)	.03 (−.03, .09)	.04 (−.02, .10)	.34 (.29, .39)	.33 (.28, .39)					
12. Maternal education^a^	−.05 (−.10, .00)	.12 (.07, .17)	.09 (.04, .15)	.03 (−.02, .08)	.13 (.07, .18)	−.04 (−.09, .01)	−.05 (−.10, .00)	−.01 (−.06, .05)	−.12 (−.17, −.06)	−.05 (−.10, .01)	−.04 (−.10, .01)	‒			
13. Annual family income^c^	−.00 (−.06, .05)	.19 (.13, .24)	.08 (.03, .13)	.03 (−.02, .08)	.16 (.11, .22)	−.01 (−.06, .05)	−.01 (−.07, .04)	.01 (−.05, .06)	−.15 (−.20, −.09)	−.15 (−.20, −.09)	−.04 (−.09, .02)	.25 (.20, .30)	‒		
14. Maternal age^a^	.04 (−.01, .09)	−.03 (−.08, .02)	.04 (−.01, .09)	−.00 (−.06, .05)	−.01 (−.07, .04)	−.01 (−.06, .04)	.00 (−.05, .05)	.04 (−.01, .10)	.03 (−.02, .08)	−.02 (−.07, .04)	.00 (−.06, .06)	.23 (.19, .28)	.12 (.07, .17)	‒	
15. Gestation duration^a^	−.02 (−.07, .03)	.07 (.02, .13)	.12 (.06, .17)	.04 (−.01, .09)	.06 (.01, .12)	.04 (−.01, .09)	−.13 (−.18, −.08)	−.12 (−.17, −.06)	−.02 (−.07, .03)	.01 (−.05, .07)	−.05 (−.11, .01)	.06 (.01, .11)	.06 (.01, .11)	−.04 (−.09, .01)	‒
16. Birthweight^a^	−.02 (−.07, .03)	.06 (.00, .11)	.08 (.03, .13)	.06 (.01, .12)	−.00 (−.06, .05)	−.08 (−.13, −.03)	−.06 (−.11, −.01)	−.05 (−.11, .00)	.02 (−.03, .08)	.01 (−.04, .07)	−.04 (−.10, .01)	.02 (−.03, .07)	.01 (−.04, .07)	−.01 (−.06, .04)	.56 (.52, .59)

Note. BSID-III, Bayley Scales of Infant and Toddler Development–Third Edition; MCDI, MacArthur-Bates Communicative Development Inventories. Values in brackets indicate the 95% confidence interval for each correlation.

^a^ Pearson correlations.

^b^ 0 = boy, 1 = girl.

^c^ Spearman correlations.

Results of multiple linear regressions are presented in Table 3. There were no multivariable extreme data, and all assumptions were met. Antidepressant use was related to lower BSID-III fine motor score after adjusting for confounders (*β* = −0.26), and the strength of this association was slightly modified after inclusion of depressive symptoms (*β* = −0.29), maternal perceived stress (*β* = −0.23), but not when both were included (*β* = −0.26). Negative standardised linear regression coefficients with BSID-III gross motor score were in the same range although slightly lower. BSID-III cognitive and MCDI language development scores were unrelated to antidepressant use. The E-value has been estimated to address unmeasured confounding in the fully adjusted models. In the estimation of the association between exposure to antidepressants and fine motor development, the observed association could be explained away by an unmeasured confounder which would be associated with both the exposure and the developmental outcome by a risk ratio between 1.18 and 1.86-fold. As a comparison, all potential confounders included in our models have a combined E-value of 1.16.

**TABLE 3:** Standardized linear regression coefficients of exposure to antidepressants (yes/no) on child’s development.

	Fine motor (*n* = 1,397)	Gross motor (*n* = 1,397)	Cognition (*n* = 1,456)	Language (*n* = 1,055)
	β (95% CI)	β (95% CI)	β (95% CI)	β (95% CI)
Model 1	−0.26 (−0.50, −0.02)	−0.23 (−0.48, 0.02)	−0.14 (−0.38, 0.09)	0.11 (−0.21, 0.45)
Model 2	−0.29 (−0.53, −0.06)	−0.25 (−0.50, −0.00)	−0.13 (−0.38, 0.09)	0.15 (−0.17, 0.48)
Model 3	−0.23 (−0.47, 0.02)	−0.22 (−0.48, 0.03)	−0.09 (−0.33, 0.15)	0.16 (−0.16, 0.49)
Model 4	−0.26 (−0.52, −0.02)	−0.24 (−0.49, 0.01)	−0.10 (−0.34, 0.14)	0.18 (−0.15, 0.50)

Note. All models included child’s sex and age at testing, maternal education and age, and annual family income. In Model 2, prenatal depressive symptoms (first trimester) was added to Model 1, while in Model 3, prenatal maternal stress (second trimester) was added to Model 1. In Model 4, both prenatal stress and prenatal depressive symptoms were added to Model 1.

Results of mediation analyses shows that all indirect effects were non-significant (see Supplementary Figures S4–S11). No differences according to child sex were found in moderation analyses (data not shown).

## Comment

### Principal findings

We report negative associations between prenatal antidepressant exposure and fine and gross motor development, but no association with cognitive and language development. Inclusion of prenatal maternal distress only slightly attenuated the strength of these associations. These negative associations are of low magnitude since changes in BSID-III scores were only about one point in relation to exposure. No indirect association, mediated by gestation duration or birthweight, were found, nor differential effects according to child sex.

### Strengths of the study

The sample size of this study is greater than most of the published studies to date, thus allowing for better statistical power. Among the 17 studies consulted, 13 included a sample of less than 800 participants (Nulman et al., 1997; Nulman et al., 2002; Simon et al., 2002; Casper et al., 2011; Gentile and Galbally, 2011; Nulman et al., 2012; Austin et al., 2013; Casper et al., 2003; Santucci et al., 2014; Johnson et al., 2016; Gustafsson et al., 2018; van der Veere et al., 2020; Galbally et al., 2021). We used prospective data providing antidepressant use and maternal distress at each trimester of pregnancy. The adjustment for prenatal distress addresses a major limitation of previous studies. Furthermore, our results appear to be robust against unmeasured confounding: the E-value analysis indicated that an unmeasured confounder would need to have a stronger association with exposure and developmental outcomes than all our covariates combined, which is unlikely since we controlled for well recognized and empirically supported confounding variables. Finally, the validated tool used to assess child cognitive, fine and gross motor development provided objective information independent from parental reports.

### Limitations of the study

Antidepressant use during pregnancy was low (*n* = 61, 4.1%) in this study sample, which makes impossible to consider dose, duration, classes of antidepressants and timing of exposure in the analyses. Women who used antidepressants during pregnancy took them daily for most of their pregnancy. The medication log was not specific to antidepressants, and we assumed that if no antidepressant agents were reported, none were taken. Under-reporting due to social desirability could potentially lead to misclassifications in the non-exposed group, which may have reduced the strength of the negative associations reported. Finally, the assessment of language development only considered vocabulary knowledge, which do not represent the multidimensional aspect of language development, and, maternal report of child vocabulary may be prone to bias such maternal education, distress and use of antidepressant. Additionally, the testing of vocabulary in young children could strongly depend on the education/training given by parents and thus may not fully reflect the underlying deficits of the developing brain. One major limitation is the uncertainty regarding MCDI, given the mother’s application.

### Interpretation

Antidepressant use during pregnancy was reported by 4.1% of the participants, which is lower than the North American prevalence (5.5%) (Molenaar et al., 2020), but similar to Quebec statistics reporting a prevalence of 3.7% during the first trimester, which later drops to less than 2% for the subsequent trimesters (Ramos et al., 2007).

Our findings indicate that antidepressants use during pregnancy is negatively related to fine and gross motor development, but not to cognitive and language development at 2 years of age, and that the associations with motor development are only slightly attenuated after consideration of prenatal maternal distress. The strength of these negative associations, in the range of −0.22 to −0.29, should be considered of low magnitude since changes in BSID-III motor scores are only about one point lower in relation to the exposure (data not shown). These results corroborate those of two other prospective cohort studies using the Ages and Stages Questionnaire (completed by the mother) and the BSID-III, and adjusting for maternal distress (Handal et al., 2016; van der Veere et al., 2020). They both adjusted for depressive symptoms and anxiety and found negative associations with child outcomes, and Van der Veere reported differences of 1.1-1.2 BSID-III gross motor score, which is comparable to the strength of associations found in our study. The strength of associations we observed (*β* from −0.22 to −0.29) are in same range of those from a meta-analysis: effect size of 0.22 (95% CI 0.07, 0.37) (Grove et al., 2018).

In our study, adjusting for depressive symptoms did not change the strength of the associations between antidepressant and motor development since depressive symptoms were unrelated to motor development scores, most likely due to the objective assessment of motor development instead of relying on maternal report. The strength of the antidepressant-fine motor association was only slightly reduced after adjusting for maternal perceived stress (from −0.26 to −0.23) and remained the same for gross motor development (−0.23 to −0.22). We expected a greater decrease in the antidepressant-outcome association considering known associations of prenatal perceived stress with foetal brain development (Wu et al., 2022) and child development (D'Souza et al., 2019; Polanska et al., 2017; Berthelon et al., 2021). This could be due to relatively low levels of maternal prenatal perceived stress (median PSS-4 score of 3 out of a potential maximum score of 16) in this economically advantaged sample, largely composed of mothers in couples (96%) who graduated from university.

The absence of an association between prenatal exposure to antidepressants and cognitive development at age 2 years is consistent with the current literature. Except for one prospective cohort study, which reported a mean difference of −0.8 points on BSID-III for exposed children (yes/no) after adjustment for depressive symptoms and anxiety (van der Veere et al., 2020), all other studies adjusting for prenatal maternal distress (depressive symptoms and/or anxiety) found no evidence of associations between prenatal exposure to antidepressants (yes/no, or duration of exposure for Johnson et al.) and cognition, as assessed with objective tools (e.g., BSID-II, Differential Ability Scales, McCarthy Scales of Children’s Abilities, Wechsler Preschool and Primary Scale of Intelligence) (Nulman et al., 2002; Nulman et al., 2012; Johnson et al., 2016; Gustafsson et al., 2018). In the present study, adjusting for depressive symptoms and maternal perceived stress did not modify the strength of the associations.

For language development, we found conflicting results in the literature with studies reporting negative (Skurtveit et al., 2014; Johnson et al., 2016) and positive associations (Nulman et al., 2002). Johnson et al. found negative associations in a sample of 178 participants (102 exposed), using the duration of exposure and language development assessed by an objective tool independent from maternal report, while adjusting for depressive symptoms (Johnson et al., 2016). Skurtveit et al. also found negative associations, using data on 51,748 participants (386 exposed), with language development as reported by the mother, while adjusting for depressive symptoms and anxiety (Skurtveit et al., 2014). On the other hand, Nulman et al. found positive associations with language assessed by an objective tool in a study group of 122 participants (86 exposed), while adjusting for depressive symptoms (Nulman et al., 2002). In the present study, we reported positive regression coefficients with large confidence intervals indicative of no associations. The instrument used was completed by the mother and the results are thus likely influenced by her subjectivity and moods. Negative associations between language scores and maternal perceived stress were indeed observed. Also, some children were exposed to more than one language, but evaluated only in one. Because we cannot be confident that the child’s language score truly represents linguistic abilities in these family situations, we limited our analyses to the sample of children exposed at least 70% of the time to the language of testing. This may have reduced the language score variability.

The absence of indirect effects in this study is most likely explained by the absence of associations between antidepressant exposure and the chosen mediators, gestation duration, and birthweight, although small associations are seen between these mediators and the developmental outcomes. Even if duration of gestation ≤37 weeks has been associated with antidepressant use during pregnancy, when studies adjust for depression, these associations are diminished, or even nullified (Tak et al., 2017). With regard to birthweight, the literature is inconsistent about its relationship with prenatal antidepressant exposure, but some systematic reviews report an absence of significant associations (Prady et al., 2018; Tak et al., 2017).

Finally, we found no differential effects depending on the child’s sex. Very few studies commented on sex dependant effects. Pedersen and its colleagues did report that boys were at greater risk for motor development delay, but the associations reported were non-significant (Pedersen et al., 2010).

## Conclusion

This study provides evidence of negative associations between antidepressant use during pregnancy and subsequent motor development among 2 years old children. However, the effect sizes remain very small. No associations are reported with cognitive and language development. Even if adjustment for maternal depressive symptoms and prenatal perceived stress only slightly modified the antidepressant-motor development associations in our study, it remains important to consider these variables in other population studies providing larger samples and greater statistical power. Such larger size studies would also allow to consider dose, duration and classes of antidepressants, timing of exposure during the prenatal period, and to study whether obstetric complications may be considered as a mediator or a moderator of the relationship between prenatal exposure to antidepressant and later child development.

## Data Availability

The datasets presented in this article are not readily available because any researcher interested in accessing the IRNPQEO Biobank and Database can contact the Biobank Manager. Requests to access the datasets should be directed to https://www.irnpqeo.ca/en/―email address of Biobank Manager: isabelle.krauss.hsj@ssss.gouv.qc.ca.
