# Supplementary material for: Antidepressants use during pregnancy and child psychomotor, cognitive and language development at 2 years of age—Results from the 3D Cohort Study
Source: Front Pharmacol. 2023 Nov 16;14:1252251. doi: 10.3389/fphar.2023.1252251 (PMC10687276; doi:10.3389/fphar.2023.1252251)
Supplement: Supplementary file 1 [file DataSheet1.pdf]

Supplementary material

Noémie Tanguay[1,2], Nadia Abdelouahab[3], Marie-Noelle Simard[4,5], Jean R. Séguin[4,5], Isabelle Marc[1,2], Catherine M. Herba[4,6], Andrea A.N. MacLeod[7], Yohann Courtemanche[2], William D. Fraser[4], Gina Muckle[1,2]

[1]Université Laval; [2]Centre de recherche du CHU de Québec-Université Laval; [3]Centre de recherche du CHU de Sherbrooke; [4]Centre de recherche du CHU Sainte-Justine; [5]Université de Montréal; [6]Université du Québec à Montréal;[7]University of Alberta.

Supplemental tables

Supplementary Table 1

Characteristics of the project subsample and the participants not included

Variable	Project subsample (N = 1489)		3D participants not included (N = 876)		Mean difference [*MD* (95% CI)] or χ^2 [χ^2 (*df*), *p*-value] or *U* [*p*-value]
	n	Mean ± SD or *n* (%)	n	Mean ± SD or *n* (%)	
Family and maternal characteristics					
Marital status (% married or living with someone)	1488	1427 (95.9)	874	806 (92.2)	14.45(1), <.01
Annual income (in CDN$)	1431		816		85.55(2), <.01
< 40 000$		189 (13.2)		210 (25.7)	
40 000$ - 79 999$		425 (29.7)		289 (35.4)	
≥ 80 000$		817 (57.1)		317 (38.8)	
Mother's education (years)	1463	16.85 ± 3.07	854	16.04 ± 3.56	-0.81 (-1.1, -0.53)
Parity before childbirth	1463	0.58 ± 0.81	842	0.70 ± 0.89	23.03(8), <.01
Maternal age at delivery (years)	1486	32.14 ± 4.42	749	31.62 ± 4.93	-0.51 (-0.93, -0.09)
Maternal stress during pregnancy[a]	1347	3.44 ± 2.80	478	3.81 ± 2.96	.02
Maternal depressive symptoms during pregnancy[b]	1209	7.16 ± 4.52	527	7.85 ± 5.16	0.69 (0.18, 1.19)
Maternal tobacco use during pregnancy (% yes)	1489	145 (9.7)	868	121 (13.9)	9.67(1), <.01
Maternal alcohol use during pregnancy (% yes)	1488	818 (55.0)	868	361 (41.6)	39.28(1), <.01
Prenatal exposure to antidepressant					
Maternal use (% yes)	1489	61 (4.1)	876	42 (4.8)	0.65(1), 0.42
Daily use during pregnancy (%)	53	51 (96.2)	41	38 (92.7)	0.07(1), 0.77
Duration of use (in months)	61	5.51 ± 3.20	42	6.38 ± 3.19	0.22

Variable	Project subsample (N = 1489)		3D participants not included (N = 876)		Mean difference [*MD* (95% CI)] or χ^2 [χ^2 (*df*), *p*-value] or *U* [*p*-value]
	n	Mean ± SD or *n* (%)	n	Mean ± SD or *n* (%)	
Child characteristics					
Child sex (% girls)	1489	743 (49.9)	749	372 (49.7)	0.01(1), 0.92
Gestational age (weeks)	1477	38.98 ± 1.60	743	38.40 ± 3.07	.01
Birthweight (kg)	1487	3.39 ± 0.51	747	3.22 ± 0.69	-0.16 (-0.22, -0.10)
Age at BSID-III assessment (months)	1460	24.77 ± 1.76	70	30.91 ± 4.59	< .01
Age at MCDI assessment (months)	1055	24.74 ± 1.76	51	30.35 ± 5.01	5.53 (4.11, 6.94)
Child outcomes					
Cognition raw score – BSID-III	1456	63.97 ± 5.40	65	68.31 ± 7.46	4.34 (2.47, 6.21)
Fine motor skills raw score– BSID-III	1397	41.22 ± 3.43	61	46.66 ± 5.96	5.43 (3.90, 6.97)
Gross motor skills raw score– BSID-III	1397	55.09 ± 3.65	61	58.79 ± 5.50	3.70 (2.28, 5.12)
Language raw score – MCDI	1055	55.36 ± 22.99	72	64.63 ± 27.95	11.04 (4.35, 17.73)

Note. BSID-III = Bayley Scales of Infant and Toddler Development – Third Edition; MCDI = MacArthur-Bates Communicative Development Inventories.

[a] Perceived Stress Scale – 4 items, 2nd pregnancy trimester. [b]Center for Epidemiologic Studies – Depression Scale – 10 items, 1st pregnancy trimester.

Supplemental figures

Supplementary Figure 1

Flowchart for 3D Cohort Study participants included in the analyses, depending on developmental outcome

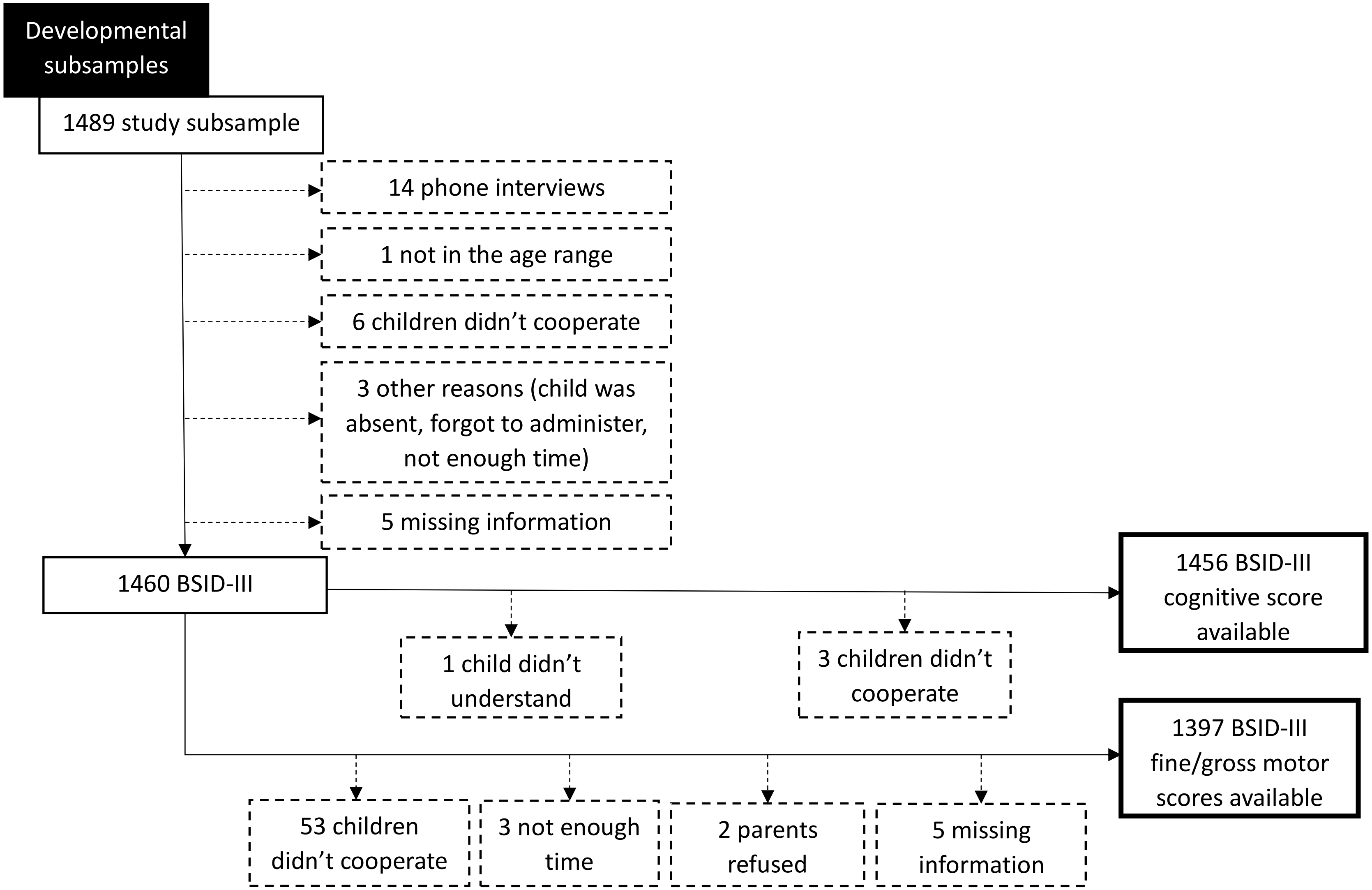

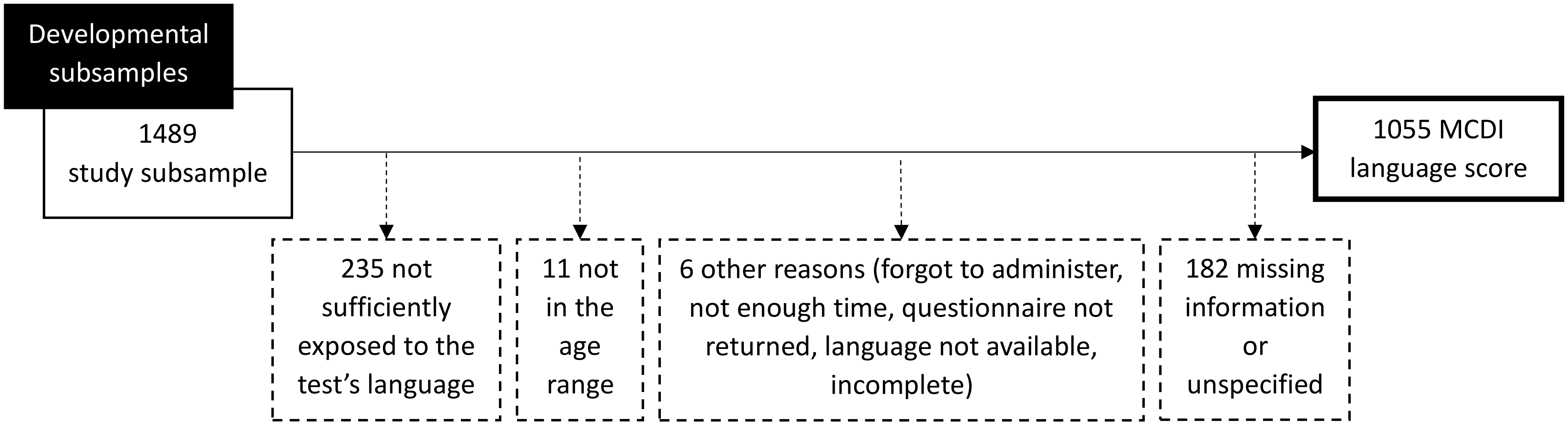
Developmental subsamples
1489 study subsample
1055 MCDI language score
235 not sufficiently exposed to the test's language
11 not in the age range
6 other reasons (forgot to administer, not enough time, questionnaire not returned, language not available, incomplete)
182 missing information or unspecified

Supplementary Figure 2

DAG used for the choice of confounding variables

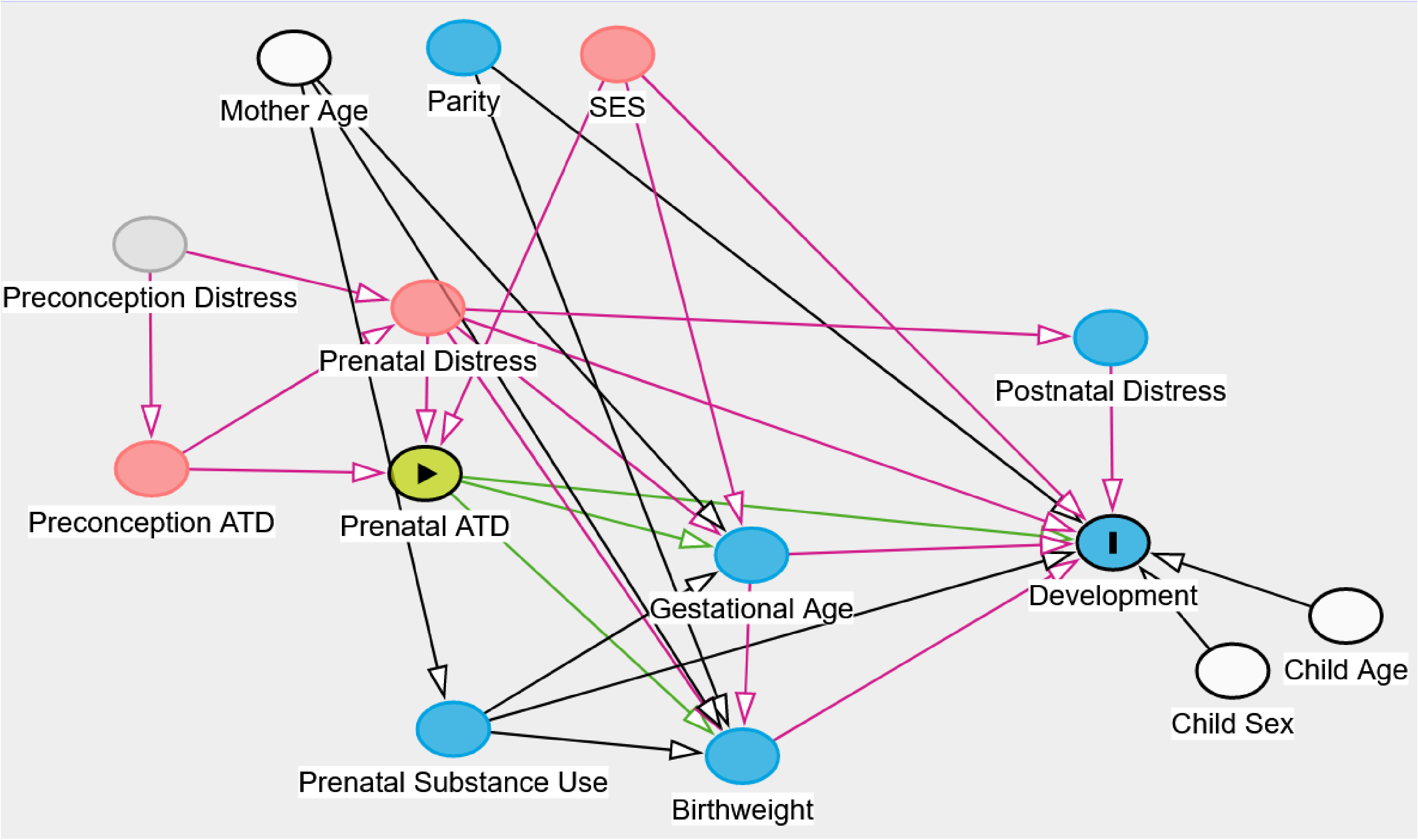

Note. ATD = Antidepressant use; SES = Socio Economic Status (including maternal education and annual income).

Supplementary Figure 3

Multivariate missing data patterns

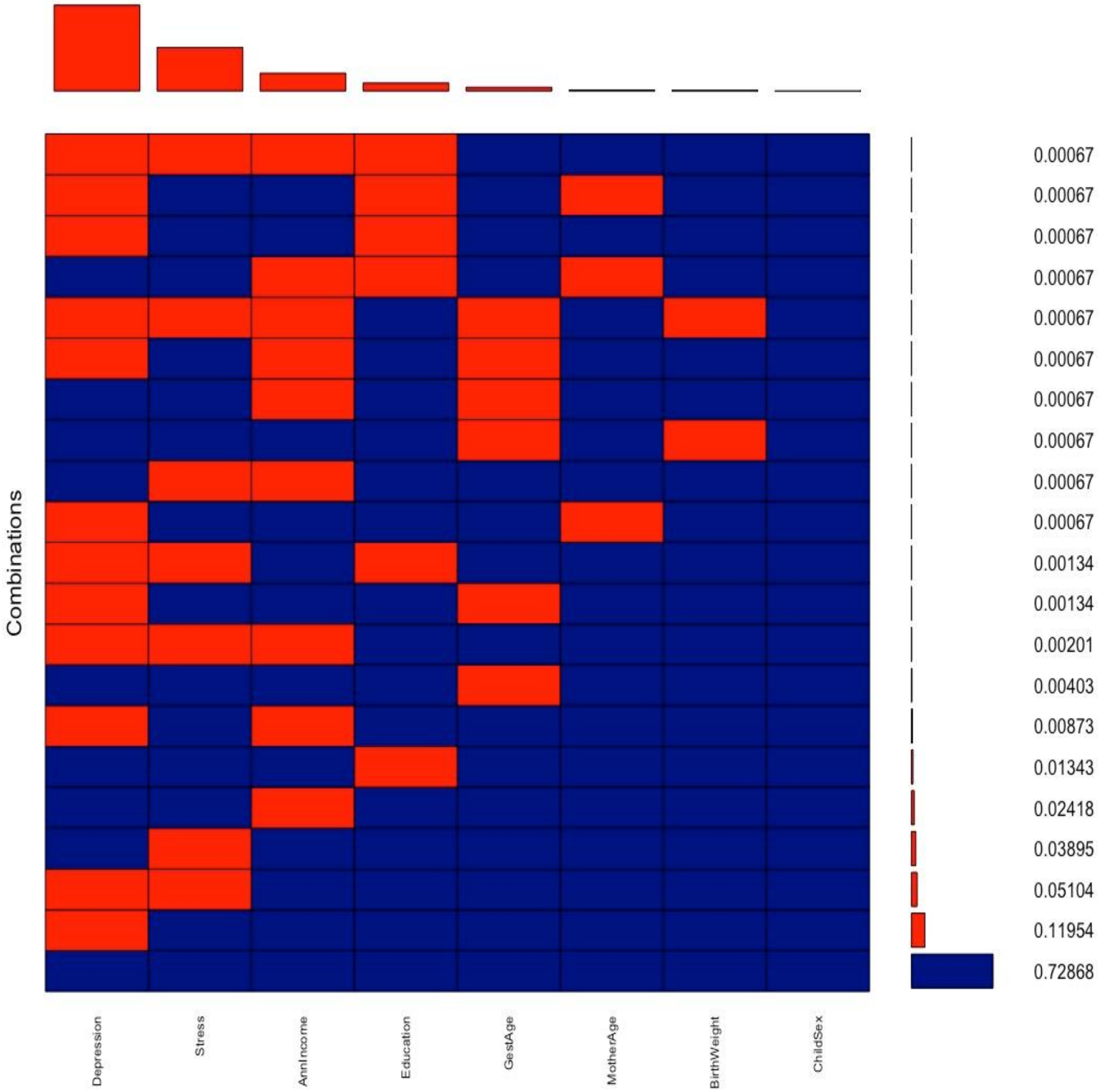

Note. Blue cells indicate available data, red cells represent missing data with each row a different data pattern. Numbers on the right-hand side of the figure are the proportion of data in each pattern. AnnIncome = Annual Income; GestAge = Gestational Age.

Supplementary Figure 4

Mediation model between antidepressant exposure and fine motor skills, explained by gestational age

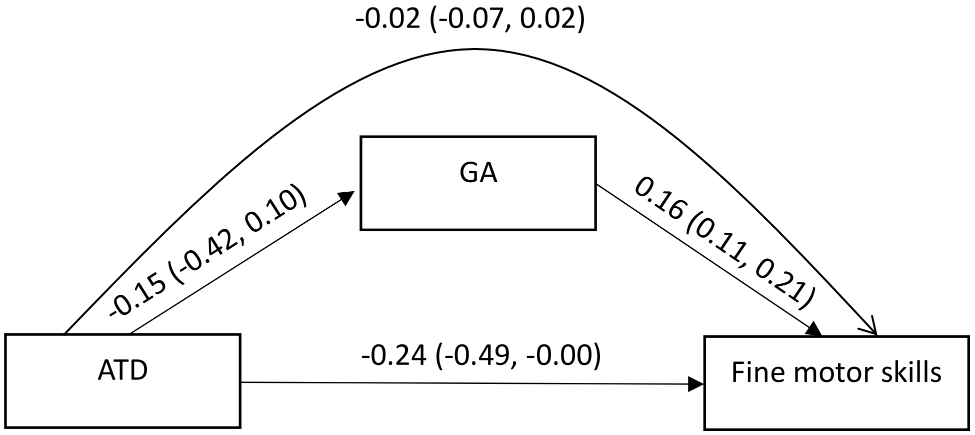

Note. Coefficients are standardized with 95% CI. ATD = exposure to antidepressants; GA = gestational age (# weeks).

Supplementary Figure 5

Mediation model between antidepressant exposure and gross motor skills, explained by gestational age

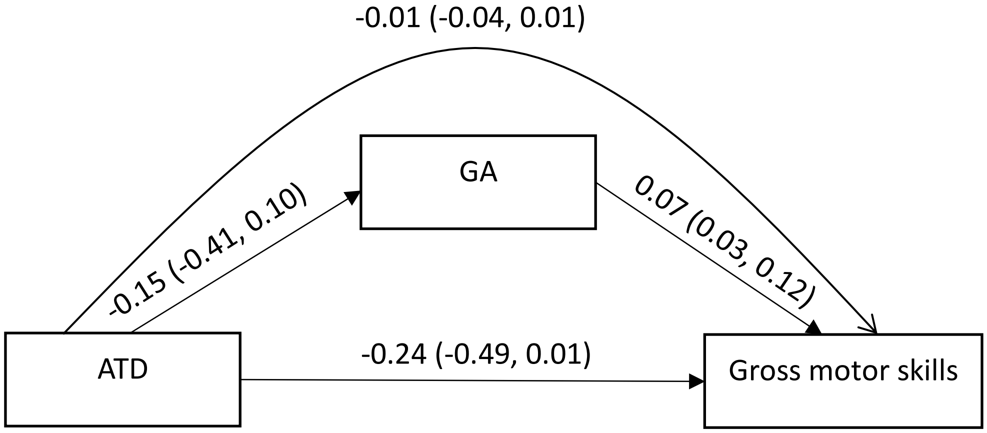

Note. Coefficients are standardized with 95% CI. ATD = exposure to antidepressants; GA = gestational age (# weeks).

Supplementary Figure 6

Mediation model between antidepressant exposure and cognition, explained by gestational age

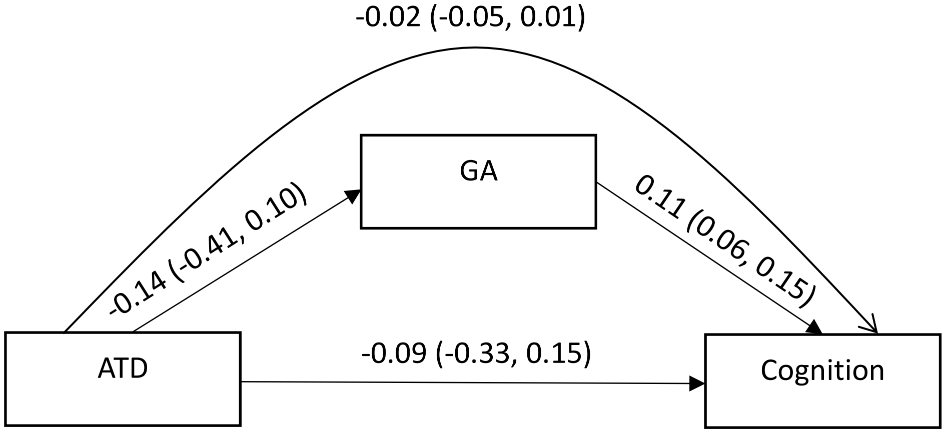

Note. Coefficients are standardized with 95% CI. ATD = exposure to antidepressants; GA = gestational age (# weeks).

Supplementary Figure 7

Mediation model between antidepressant exposure and language, explained by gestational age

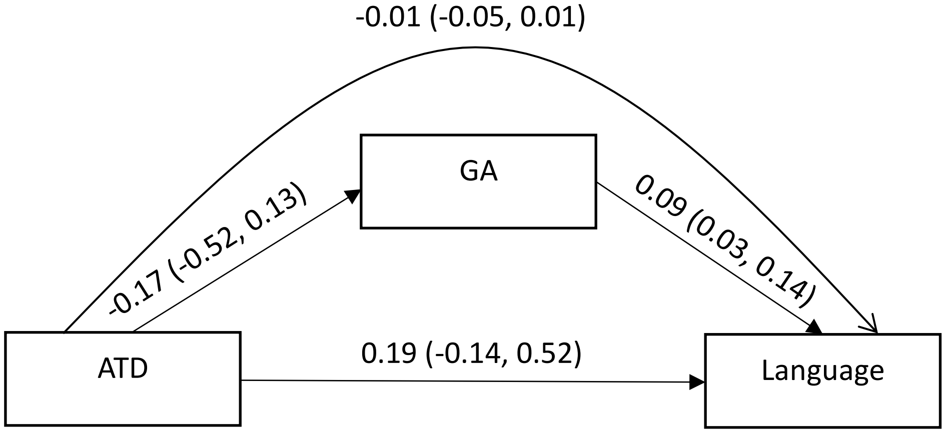

Note. Coefficients are standardized with 95% CI. ATD = exposure to antidepressants; GA = gestational age (# weeks).

Supplementary Figure 8

Mediation model between antidepressant exposure and fine motor skills, explained by birthweight

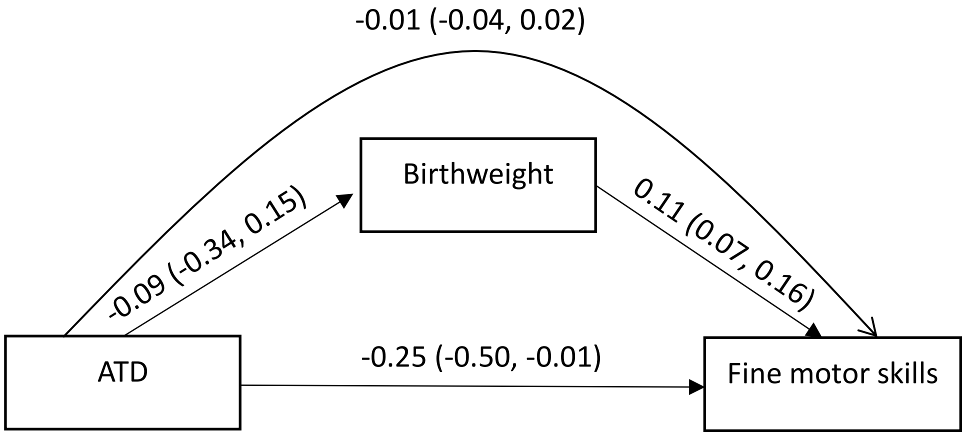

Note. Coefficients are standardized with 95% CI. ATD = exposure to antidepressants. Birthweight in grams.

Supplementary Figure 9

Mediation model between antidepressant exposure and gross motor skills, explained by birthweight

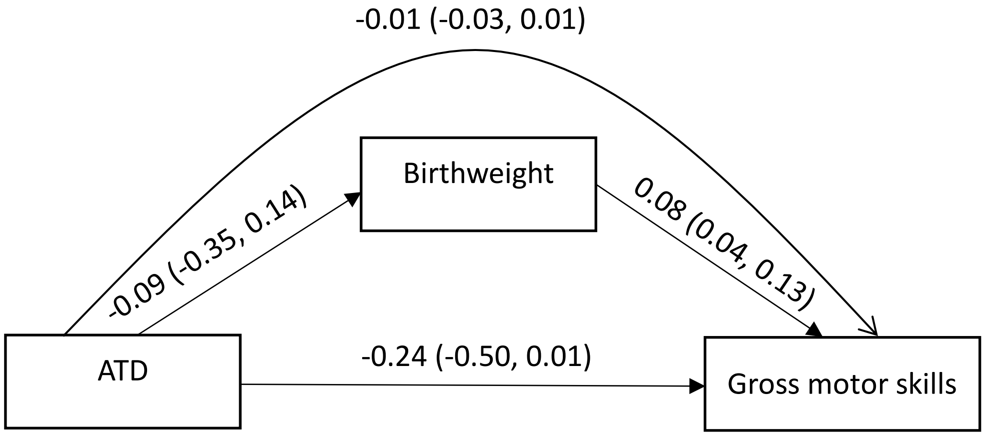

Note. Coefficients are standardized with 95% CI. ATD = exposure to antidepressants. Birthweight in grams.

Supplementary Figure 10

Mediation model between antidepressant exposure and cognition, explained by birthweight

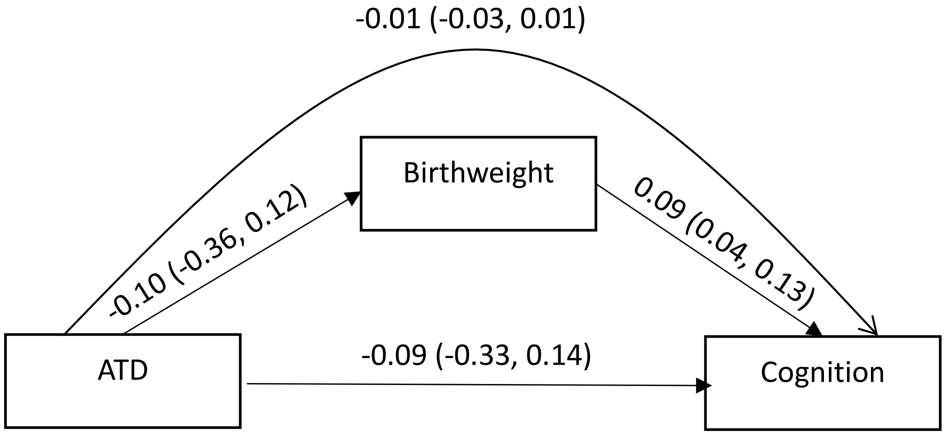

Note. Coefficients are standardized with 95% CI. ATD = exposure to antidepressants.
Birthweight in grams.

Supplementary Figure 11

Mediation model between antidepressant exposure and language, explained by birthweight

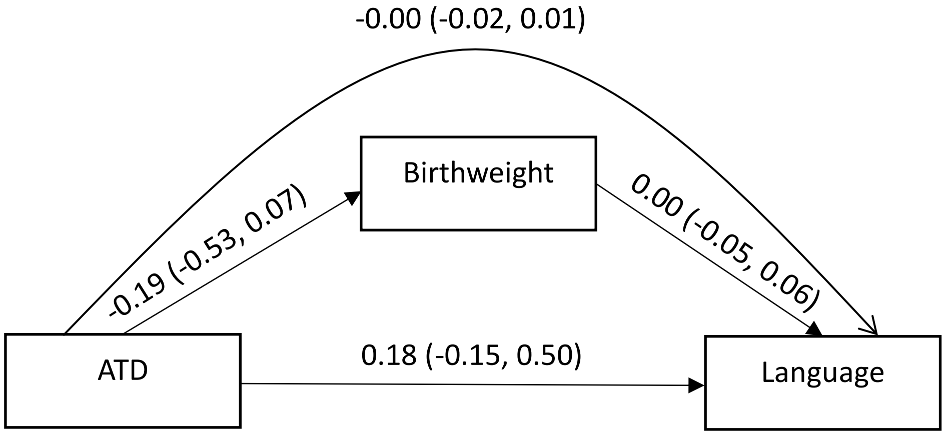

Note. Coefficients are standardized with 95% CI. ATD = exposure to antidepressants.
Birthweight in grams.
